# ME/CFS: Past, Present and Future

**DOI:** 10.3390/healthcare9080984

**Published:** 2021-08-03

**Authors:** William Weir, Nigel Speight

**Affiliations:** 1 Royal Free Hospital, London W1G 9PF, UK; 2 University Hospital of North Durham, Durham DH1 1QN, UK; speight@doctors.org.uk

**Keywords:** myalgic encephalomyelitis/chronic fatigue syndrome (ME/CFS) controversy, dogma, psychological causation

## Abstract

This review raises a number of compelling issues related to the condition of Myalgic Encephalomyelitis/Chronic Fatigue Syndrome (ME/CFS). Some historical perspective is necessary in order to highlight the nature of the controversy concerning its causation. Throughout history, a pattern tends to repeat itself when natural phenomena require explanation. Dogma usually arrives first, then it is eventually replaced by scientific understanding. The same pattern is unfolding in relation to ME/CFS, but supporters of the psychological dogma surrounding its causation remain stubbornly resistant, even in the face of compelling scientific evidence to the contrary. Acceptance of the latter is not just an academic issue; the route to proper understanding and treatment of ME/CFS is through further scientific research rather than psychological theorisation. Only then will a long-suffering patient group benefit.

The history of human civilisation is littered with examples of natural phenomena, including human disease, initially explained by dogma. The dogma is initially created to fill a void in comprehension, but it is eventually replaced by rational scientific understanding. The creators of such dogma are often authoritarian, hierarchical figures who then ferociously defend their own creation. The classic manifestation of this was what Galileo encountered when he proposed, on the basis of careful observations through the new technology of the telescope, that the planet was not the centre of the solar system. Despite the scientific validity of his observations, he was threatened with torture by the Catholic inquisition if he did not recant. He was speaking truth to a powerful establishment, and the conflict came to a head in 1633, when, under severe duress, he was forced to withdraw his heretical ideas. The Catholic church reprieved him eventually, but not until 1992. Galileo’s difficulties with the Catholic church are a good early example; he spoke scientific truth to authoritarian power and suffered the consequences.

There are similar examples of this pattern in medical history. Ignaz Semmelweis, working in an obstetric ward at the General Hospital in Vienna in 1846 noticed the large difference in mortality from puerperal fever between a ward where birthing women were attended by midwives, and another where the women were attended by doctors and medical students. The latter divided their time between the autopsy room and the ward. Semmelweis observed that the midwives washed their hands between deliveries, whereas the doctors and medical students did not, even after performing autopsies on the victims of puerperal fever. The patients attended by the doctors and medical students died, embarrassingly, more frequently from puerperal fever, and Semmelweis recognised correctly that a noxious agent was being transmitted from the autopsy room by unwashed hands. The precise cause, in the light of current scientific understanding, is now blindingly obvious. Unfortunately, for Semmelweis, this was before the discovery of disease-causing microbes. His medical colleagues, lacking Semmelweis’ insights, were greatly offended by the implication that by not washing their hands, they were somehow responsible for the excess deaths. Consequently, he was hounded out of his post. He was ahead of his time, but nowadays he can be rightly regarded as a hero, having spoken truth to power, as Galileo did, at great personal expense.

John Snow’s scientifically correct perceptions of cholera transmission were also up against a contemporary dogma. In 1855, he published his treatise, which found that the cause of cholera was spread through drinking water. What he wrote has stood the test of time and is now regarded as a model of scientific validity. He recognised that drinking water from known sources was the cause, and he was persistent enough to collect the data to prove this by undertaking painstaking house-to-house visits through the streets of London. Nonetheless, nearly 30 years elapsed before Robert Koch demonstrated the presence of *Vibrio cholerae* on the gut-lining of cholera victims at autopsy, having also been able to demonstrate the presence of this microbe in drinking water. At the time of Snow’s publication, however, the prevailing dogma was that cholera was spread by rotten smells— “miasmata”—from dead bodies and rotting vegetable matter. Predictably, prominent members of the Royal College of Physicians at the time, declared Snow’s work “untenable” because their dogma was being challenged.

This collective mindset meant that contemporary management of cholera was equally wide of the mark. Bloodletting and rectal infusions of mutton puree were among the conventional mainstream treatments. The latter was probably challenging given the profuse diarrhoea of cholera. Furthermore, contemporary survival from cholera was seen to be rather better at the London Homeopathic Hospital where patients were spared the lethally inappropriate practice of bloodletting. One of the main pathological characteristics of cholera is reduction of circulating blood volume due to the diarrhoea, causing massive salt and water depletion. Further reduction of blood volume by bloodletting certainly hastened the death of such patients.

Additional examples of the medical profession “getting it wrong” have continued since this time. General Paralysis of the Insane—a manifestation of tertiary syphilis—and multiple sclerosis were both considered to have a psychological basis [1] until the true physical basis was discovered. There are other examples: the tremor of Parkinson’s disease had been attributed to “the expression of the moralistic man’s suppressed desire to masturbate” [2] but we now know this to be untrue. More recently, the proposition that Helicobacter pylori infection could be the cause of peptic ulcers was up against the dogma that psychological stress was the major contributor. Hitherto, the presence of this strange organism in the stomach lining was regarded as insignificant, but trials with antibiotic therapy effectively refuted this idea, and the treatment of peptic ulcers was dramatically improved [3]. All of these examples illustrate a tendency to assume that, if no pathological mechanism can be demonstrated, then, by default, psychological disorder must be the problem. Inherent in such an assumption is the arrogant belief that routine laboratory tests infallibly exclude physical disorder.

The story of ME/CFS is a prime example of such dogma. Due to the fact that routine laboratory tests for the diagnosis of this condition usually produce “normal” results, the problem must be with the psyche. One of the foundation stones of this dogma was a paper published in the BMJ in 1970 in which the cause of the famous Royal Free outbreak of ME/CFS in 1955 was attributed to “mass hysteria”. The authors did not interview any of the patients, nor any of the doctors involved; nonetheless, it seemed clear to them that the outbreak was due to mass hysteria because the majority of victims were women [4]. The background to this piece of sophistry was, and remains, the fashionable medical culture of linking physical symptoms to a psychological disorder. Mind and body are highly interactive, and certainly there are conditions in which psychological distress expresses itself with physical symptoms. Even so, there are many human diseases and infirmities in which the primary driver is physical pathology, with psychology playing a minor secondary role, if at all. Nevertheless, the psychological cognoscenti have not let this principle inhibit their wide-ranging suppositions about the role of psychology in the human condition. The result has been some very wild adventures in psychological theory. For example, a famous 20th century French psychologist once suggested in all seriousness that an erect penis could be expressed algebraically as the square root of minus one [5]. To his disciples, this was “boldly transgressive thinking”, but most sane mathematicians of either sex will have been baffled, not least because in conventional mathematics, minus one does not have a square root. Nonetheless, a culture of similar nonsense has set the scene for equally fantastic theorisation concerning other manifestations of the human condition, including the cause(s) of ME/CFS.

As with previous examples of medical dogma, the belief that ME/CFS is “psychological” will eventually be consigned to the dustbin of medical history, alongside miasma theory and suchlike. Compelling evidence of physical causation is now accumulating but the authoritarian cabal who promoted the psychological dogma are even now trying to defend it in the face of irrefutable scientific evidence to the contrary. History repeats itself, to coin a phrase, given the stories of Galileo, Semmelweis and Snow, and the cabal referred to, do not yet recognise how badly placed they are in the historical narrative of ME/CFS. In some circumstances, the tendency of exponents to hold on to their dogma is reminiscent of the tenacious way conspiracy theorists are wedded to their particular false narrative. Sadly, the argument over the cause of ME/CFS would probably have remained academic but for one grim reality: treatment based on psychological dogma has damaged patients, some very severely.

Due to the fact that ME/CFS was due, amongst other things, to “abnormal illness beliefs, buried guilt and negative thoughts”, the psychological advocates have always advised treatment intended to correct disordered psychology and its presumed consequences. The muscular weakness of ME/CFS was seen as simply due to “deconditioning” because of inactivity secondary to exercise phobia. Graded Exercise Therapy (GET) was, therefore, the answer, and abnormal illness belief and exercise phobia could be managed with Cognitive Behavioural Therapy (CBT). Both of these techniques have been widely promoted, supported in particular by the PACE trial [6], an egregious and expensive exercise in scientific sophistry whose methodology was so seriously flawed that it is now used as an example of how not to conduct scientific studies [7].

The damage caused by GET, in particular, has unfortunate historical precedents. As previously stated, bloodletting was particularly dangerous for cholera; likewise GET has caused significant harm for many ME/CFS patients, frequently consigning modestly mobile patients, adults and children alike, to a prolonged, bedbound, nasogastric-tube-fed existence. If GET were a drug, it would have been banned rapidly by the appropriate regulatory body, but in the UK, there is no such regulatory body for non-pharmacological treatments. This should be within the remit of the General Medical Council, but despite one of their stated functions being to “protect patients”, many patients have been harmed in the way described.

In respect of children in the UK with ME/CFS, the psychological dogma has been particularly harmful. The UK paediatric establishment has not recognised the physical nature of the incapacity caused by ME/CFS. It has become increasingly fashionable in British paediatrics to apply the terms “Medically Unexplained Symptoms” (MUS) and “Perplexing Presentations” (PP) under the much wider umbrella of “potential Factitious Illness (FII)”, on the specious grounds that if the doctor concerned cannot make a diagnosis, it is likely that the mother is “colluding” with her child’s symptoms. Families of children with ME/CFS are particularly at risk of being trapped in such accusations, due to the dogma-led belief in psychological disorder when all routine tests are normal. As a result of this, children with ME/CFS have sometimes been removed by social services from the security of their own home. This can then be followed by grotesquely inappropriate treatment, one extreme example of which involved a severely impaired 12-year-old boy being left unsupported, deliberately, in a hydrotherapy pool. The intention being to force him to swim, thus revealing that he was physically unimpaired and had to overcome his abnormal illness beliefs and negative thoughts about his true physical capabilities. In reality he was so physically weak that he nearly drowned, unwittingly re-enacting the medieval test for witchcraft.

There are other examples in which non-existent psychological disorder was suspected: a teenage girl with severe ME/CFS was once visited at home by her GP. He said, “Now we are going to get to the bottom of the secret phobias that are causing your illness”. The girl answered, “but I don’t have any secret phobias”, to which the doctor replied, “that’s the thing about secret phobias, you don’t know you’ve got them until we dig deep enough.” In some egregious instances, children with ME/CFS whose condition predictably worsens with GET become bedbound. They then have an alternative diagnostic label applied, such as “Pervasive Refusal Syndrome.” The skewed logic being that GET always helps ME/CFS; if it does not, the initial diagnosis of ME/CFS must have been wrong.

Mention has already been made of recognisable pathological abnormalities in ME/CFS, effectively rebutting the dogma of psychological causation. Even now the aforementioned authoritarian cabal continue to ignore or possibly regard such abnormalities as “downstream” of primary psychological disorder. Abnormalities of muscle metabolism in ME/CFS patients have now been clearly recognised, providing scientific insight into the characteristic intolerance of exercise [8,9]. The ME/CFS dogma attributes this to psychological causes, particularly “exercise phobia”. It is now evident that calibrated exercise on a bicycle ergometer on two consecutive days indicates clear differences in muscle metabolism between ME/CFS patients and healthy but sedentary, i.e., deconditioned, controls. In the ME/CFS patients, the “anaerobic threshold” decreases on the second exercise day, whereas it increases in the controls as part of the process leading to increasing physical fitness [8,9].

In lay terms, the anaerobic threshold is the point at which muscles, exercising at maximum, switch to a metabolic pathway that does not use oxygen. This allows for a final burst of energy, followed within a few seconds by a sensation of exhaustion. High anaerobic thresholds are characteristic of athletes, particularly those undertaking endurance events that enable them to run long distances without hitting their anaerobic threshold. In non-athletic, but healthy people, repeated daily exercise causes the anaerobic threshold to rise, the result being increasing physical fitness. This does not happen in ME/CFS, and misguided attempts to force exercise on the patient has exactly the opposite effect for the reasons stated above. It is highly likely that such exercise on consecutive days will lower the anaerobic threshold even further. In badly affected patients, the effect of an extremely low anaerobic threshold is severe exercise intolerance, which manifests as profound exhaustion, even with the minimal effort of getting out of bed, or such activities as eating and swallowing. Such cases often arise as a consequence of enforced exercise, unwittingly and progressively lowering the anaerobic threshold, rendering a moderately affected and previously mobile patient even more exhausted. The result is a bedbound existence for prolonged periods, some even requiring tube-feeding because the level of exhaustion is such that chewing and swallowing a normal diet becomes physically impossible.

Studies in vitro of biopsied muscle from ME/CFS patients have shown metabolic defects that underpin the findings described above. Repeated electrical stimulation of isolated muscle fibres from ME/CFS patients reveals impairments of metabolism that are not seen in healthy controls [10]. Biopsied muscle is self-evidently separate from the owner’s psyche, safely excluding any influence from this source. There are other studies that further demonstrate the physical basis of ME/CFS. Disorder of the hypothalamic/pituitary/adrenal axis (HPAA) has been recognised for at least 30 years [11,12,13,14,15,16] and may well be due to autoimmunity [17]. Reduced circulating cortisol levels are the result, with a similar reduction of HPAA responses to stresses, both physical and psychological [14]. As a consequence of this, long-standing ME/CFS patients, due to impaired ACTH output, have been shown to have significantly smaller adrenals compared to normal controls [18], and also a low circulating blood volume [19]. The latter is very likely to contribute to Postural Orthostatic Tachycardia Syndrome (POTS), a common complication of ME/CFS [19].

Immunological dysfunction is also a universal feature. Many patients, previously healthy, experience an acute infection at the onset of their ME/CFS. This can either be viral, bacterial or protozoan. The common denominator is clearly an immunological stimulus, a principle supported by the recognition that vaccination can play the same role for some. In healthy people an immune response is stimulated by the infection/vaccine, the response then shutting down when the infection/vaccine is cleared. The shutdown is due to a series of progressive checks and balances that operate efficiently in normal health. In ME/CFS, this does not happen, and immunological activity continues for reasons that are yet to be fully understood. The simplest analogy is that of a revolving door continuing to revolve with the exit blocked. Chronic inflammation is the sequel [20,21], with some researchers describing the immune system as “derailed” [22]. The resulting inflammatory process includes the brain, giving pathological validity to the term myalgic encephalomyelitis [23,24].

In conclusion, proper scientific research into the physical cause(s) of ME/CFS will eventually replace the damaging influence of pseudoscientific, psychological dogma. A reliable biomarker currently in development [25] is a big step in this direction. Also, the current Covid19 pandemic may be a cloud with a silver lining. “LongCovid”, a devastating aftermath of Covid19 infection, is currently attracting research funding. The clinical presentations of “LongCovid” are strikingly similar to those of ME/CFS, and the underlying pathology may well be the same [26]. Hopefully, the funds referred to will be used for properly directed scientific searches for the precise cause of this pathology, rather than for a PACE mark 2. To paraphrase Albert Einstein: “the definition of insanity is to do the same thing again, expecting a different result”. If sanity prevails, properly focussed scientific research will eventually bring much needed relief to a population of patients who have hitherto been very poorly served by the medical profession.
